# Applications of 3D Bioprinting Technology in Induced Pluripotent Stem Cells-Based Tissue Engineering

**DOI:** 10.3390/mi13020155

**Published:** 2022-01-20

**Authors:** Arvind Kumar Shukla, Ge Gao, Byoung Soo Kim

**Affiliations:** 1 School of Biomedical Convergence Engineering, Pusan National University, Yangsan 50612, Korea; arvindkumarshuklapnu@gmail.com; 2 Institute of Engineering Medicine, Beijing Institute of Technology, Beijing 100081, China; 3 Department of Medical Technology, Beijing Institute of Technology, Beijing 100081, China

**Keywords:** induced pluripotent stem cells (iPSCs), 3D bioprinting

## Abstract

Induced pluripotent stem cells (iPSCs) are essentially produced by the genetic reprogramming of adult cells. Moreover, iPSC technology prevents the genetic manipulation of embryos. Hence, with the ensured element of safety, they rarely cause ethical concerns when utilized in tissue engineering. Several cumulative outcomes have demonstrated the functional superiority and potency of iPSCs in advanced regenerative medicine. Recently, an emerging trend in 3D bioprinting technology has been a more comprehensive approach to iPSC-based tissue engineering. The principal aim of this review is to provide an understanding of the applications of 3D bioprinting in iPSC-based tissue engineering. This review discusses the generation of iPSCs based on their distinct purpose, divided into two categories: (1) undifferentiated iPSCs applied with 3D bioprinting; (2) differentiated iPSCs applied with 3D bioprinting. Their significant potential is analyzed. Lastly, various applications for engineering tissues and organs have been introduced and discussed in detail.

## 1. Introduction

Tissue engineering involves delivering specific cells or cell products to injured tissues or organs to restore tissue and organ function. In this field, stem cells have the potential to significantly alter the perspective of tissue engineering given their ability to self-renew and differentiate into various cellular types [1,2]. Several stem cell-based studies have demonstrated in detail that the associated anti-inflammatory, trophic, paracrine, and immune-modulatory functions of these cells could provide significant therapeutic benefits [3]; however, this is not ideal in the treatment of diseases. The combination of stem cell and tissue engineering techniques overcomes the current limitations of stem cells in human disease therapy. In this respect, advances in stem cell technology combined with tissue engineering have opened new avenues of producing functional substitutes for regenerative medicine.

In particular, the use of induced pluripotent stem cells (iPSCs) has certain advantages in stem cell therapy and tissue engineering. As adult somatic cells are used in the generation of iPSCs, the ethical dilemmas associated with the use of embryonic stem cells (ESCs) are averted. Additionally, they are easily harvested from cutaneous sources, such as skin fibroblasts, while obviating the need for invasive harvesting procedures, such as bone marrow or adipose tissue biopsies. Furthermore, iPSCs are pluripotent and thus can be differentiated into any adult cell type, enhancing their potential in the modeling of various disease processes. In principle, as iPSCs can be derived from any adult tissue, including skin, the potential pool of source cells is many orders of magnitude greater than that of other stem cell types. Finally, iPSCs can be transplanted in an autologous fashion to avoid immunogenicity and enhance their in vivo survival. Many studies have demonstrated that human iPSCs can differentiate into various lineages, including cardiomyocytes [4], neurons [5], hematopoietic progenitors [6,7], endothelial cells [8], osteoclasts [9], hepatocyte-like cells [10], and retinal cells [11,12,13,14,15,16]. Due to these distinctive advantages, iPSCs are regarded as promising candidates for cell therapy, tissue engineering, and iPSC-based disease modeling.

Despite such wide-ranging potential, technological limitations still exist in the recapitulation of 3D native tissues/organs within a hierarchical organization [17]. It has been reported that structural mimicry matches the functional characteristics of native tissues/organs, and fabricating in vivo-like structures is a crucial factor in successful tissue engineering [18]. A powerful technological platform that has emerged is 3D bioprinting due to its ability to precisely deposit various cells and biomaterials at a predefined location [19,20]. As a source of printable biomaterials, bioinks are used to produce engineered/artificial live tissue using 3D printing. These inks are mostly composed of the cells and supporting biomolecules. The combination of cells and usually biopolymer gels are defined as a bioink. Recently, many 3D bioprinting approaches and strategies in conjunction with iPSC technology have been explored with the intention of engineering more functionalized tissue/organ analogs for improved regenerative medicine [21,22]. Therefore, this review focuses on a comprehensive understanding on the usage of iPSCs and 3D bioprinting technology in advanced tissue engineering.

First, the fundamental considerations in using iPSCs are discussed, followed by the working principles of 3D bioprinting. Various bio-fabrication factors/strategies prior to 3D bioprinting are discussed. Several applications of iPSC-based 3D bioprinting are summarized by dividing them into two categories: undifferentiated iPSCs with 3D bioprinting and differentiated iPSCs with 3D bioprinting. Finally, a summary of the findings is provided.

## 2. Fundamental Considerations for the Use of Induced Pluripotent Stem Cells (iPSCs) in Tissue Engineering

### 2.1. Generation of iPSCs

**Figure 1:**
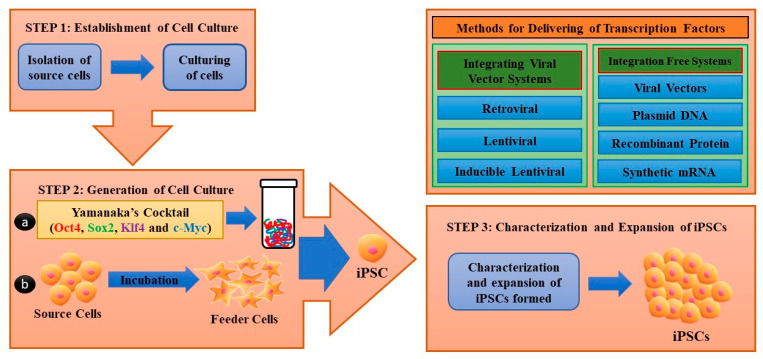
Schema for induced pluripotent stem cell (iPSC) generation. Step 1: Establishment of cell culture: source cells were cultured for further use as host cells for the delivery of reprogramming proteins. Step 2: Cultured source cells were then transfected with four factors from Yamanaka’s cocktail and incubated on feeder layers that provide nourishment to host cells and are responsible for formation of extracellular matrix under suitable media conditions. Two methods can be used for the delivery of reprogramming factors into somatic cells: integrating viral vector systems and non-integrating methods. Step 3: After iPSC formation, they were characterized by different morphological and physicochemical analyses, followed by iPSC expansion.

iPSCs can be generated directly from somatic cells by applying suitable reprogramming factors [23]. The original set of reprogramming factors are transcription factors such as Oct4 (Pou5f1), Sox2, Klf4, and cMyc [24]. While this combination of factors is the conventional method of producing iPSCs, each factor has been functionally replaced recently by related transcription factors, miRNAs, small molecules, or even non-related genes such as lineage specifiers. The generation of iPSCs utilizing several conventional cell sources, including fibroblasts, peripheral blood, and cord blood has been vigorously reported (Table 1). Several studies have demonstrated that iPSCs can be generated in three core steps. The first step was to establish an initial cell culture, the second step was to induce iPSCs, and the third step was to characterize and expand iPSCs (Figure 1). Various cell types have been produced using iPSCs (Table 2). Specifically, cell sources were first isolated and reprogramming factors were subsequently added to induce the transfected cells. Two different transfection methods include integrating methods involving lentiviral, inducible lentiviral, and retroviral vectors, and non-integrating methods involving non-integrating viral vectors, such as adenovirus and Sendai virus, transgenes, plasmid DNA transfer, synthetic mRNA, and recombinant proteins (Table 3). These transfected cells were then incubated under feeder layers (fibroblasts and keratinocytes, similar to several types of cells known as feeder cells) with the culture medium containing reprogramming factors. Morphological and physicochemical analyses were performed to observe the significant characteristics of the iPSC colonies. Notably, the cellular morphology of the iPSCs can be verified based on their round shape, large nucleus, and low cytoplasm. In general, reprogrammed cell colonies are self-renewing, tightly packed, flat, sharply edged, and extremely mitotically active. Moreover, successful generation of iPSCs can be studied through the expression of several cell surface proteins, including SAS-4, alkaline phosphatase, and transcription factors Oct4, Sox2, and Nanog.

### 2.2. Brief Discussion on the Advantages and Disadvantages of iPSCs

Currently, with the extensive advances in scientific research into stem cells, iPSCs have evolved as a viable therapeutic alternative to deflect the ethical problems associated with ESCs. In comparison to gene therapy, iPSCs provide numerous advantages in using nuclear transfer or embryonic cell-mediated gene therapy. Given that iPSCs are generated using somatic cells sourced from the patient’s body, they cause no adverse reaction from the patient’s immune system. Additionally, iPSCs can be utilized as screening tools during the discovery of new drugs to predict the toxicity and optimal therapeutic drug responses in human tissues/organs. Furthermore, iPSC-based disease models could be advantageous in developing personalized medicine in various areas of disease management. To obtain the maximum benefit from iPSCs in disease modeling, researchers are now focusing on aging, maturation, and metabolism to recapitulate the pathological features observed in patients [54]. Compared to pediatric disease modeling, adult-onset disease modeling using iPSCs requires proper maturation for the full manifestation of pathological features. Further advances in iPSC technology can be utilized for patient-specific drug treatment, maturation-based disease modeling, and alternative approaches to compensate for the current limitations in patient iPSC modeling.

Quality control is essential to effective production. First, the selection of either the autologous or allogeneic option is crucial. Autologous treatment should be designed to control variations in the quality, consistency, and safety of generated iPSCs with respect to age, health, and gender. It is essential to conduct long-term research and evaluation on tumor growth and immune responses in patients with iPSCs. Allogeneic treatment was well characterized by iPSCs, and then it was stored in cell banks for therapeutic implementation. Donors must satisfy the conditions for eligibility, and the donor must be aware that their collected sample can be used or stored after testing while meeting all ethical guidelines. After the collection of donor cells, they were cultured and selected for appropriate reprogramming methods. The application of retroviral vectors carries the risk of genetic mutations that cause cancer. Therefore, they are not suitable for therapeutic applications. To address this issue, non-viral methods are used, and the specific method is selected based on its corresponding advantages and disadvantages. After generating iPSCs, appropriate characterization is essential. Maturation is required to confirm that the generated iPSCs are functionally pluripotent by the expression of several pluripotent markers; the capacity of these iPSCs to differentiate into all three germ layers has also been confirmed. After confirming pluripotency, differentiation capacity, and efficiency of iPSC generation, they were approved for clinical therapeutic application. The generated cells were functionally evaluated by comparing them with the physiological characteristics of iPSCs generated from ESCs.

The end product is influenced by the quality of the initial stage material utilized as a cell source and the qualitative and quantitative variation in other raw materials utilized during iPSC generation. The identification and control of these parameters are required to standardize every production process to ensure reproducible and consistent output is established in accordance with the standards. Manual methods studied in the generation of iPSCs may not be appropriate for the production of large number of cells. It is essential to plan the development of quality control regulations pertaining to end-product preparation methods. Therefore, an efficient method is required to detect contamination during the administration of new cells. A backup plan must be prepared should the sterility test fail for any reason and to ensure that the product has been administered to the patient. To ensure patient safety, a quality test should be performed to verify that the final cell therapy fulfills all the required criteria. The critical advantages and disadvantages associated with the usage of iPSCs are summarized in Table 4.

## 3. Three-Dimensional (3D) Bioprinting Techniques Integrated with iPSCs Technology

Three-dimensional (3D) bioprinting is a modern technology that enables the construction of precisely controlled 3D tissue structures by evaluating cell characterization with biomolecules similar to the extracellular microenvironment of the tissue [69,70]. The iPSC-based translational study demonstrated the synergetic benefit of stem cell research and 3D-biomaterial engineering. The efficacy of 3D tissue/organ structure printing has improved because of the rapid development of functional bioinks that are printable with hydrogels encapsulating living cells. For example, 3D bio-printed cartilage and articular bone, glioma cell-laden scaffolds, and cardiac patches have been fabricated [71,72,73]. As a result, combining 3D scaffolds with 3D bioprinting technologies may help maintain the proper cellular microenvironment, including cell proliferation and survival and effective integration into living tissues. Moreover, the effective application of 3D bioprinting structures involves the fabrication of bone, skin, vascular grafts, and heart valves used for improvements, including improved mechanical strength. For effective 3D bioprinting, in depth knowledge of human anatomy, histology, physiology of specific tissue/organ organization, and the microenvironment (comprising various types of cells) is required. These are the essential characteristics considered during tissue/organ repair and regeneration. These methods have a plethora of possible applications; however, we are only exploring some of the possibilities. The safer application of iPSC-derived 3D bioprinted tissues/organs will enable the fabrication of specific tissues or organs that will be utilized for tissue engineering in the future [74,75].

**Figure 2:**
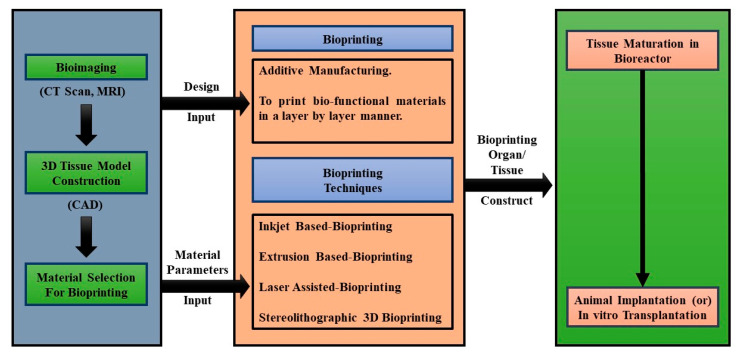
Flow diagram of 3D bioprinting process.

The 3D bioprinting process comprises three different stages: pre-fabrication, fabrication, and post-fabrication stages. The first stage involves planning all the details that lead to the bioprinting process. This stage includes the imaging process, performed by MRI, CT, and other imaging methods, to evaluate the structural anatomy and specific function of a tissue. Next, CAD methods translate the analyzed image data into the layout of the 3D bioprinting fabrication [76]. The second stage involves the construction and manufacturing of 3D bioprinted tissues. In this stage, various difficulties arise when selecting specific printing materials, such as bioinks, scaffolds, and other important additives (Figure 2). Finally, the third stage constitutes the outputs from the earlier stages ready for in vivo application (Table 2 and Table 3) [77].

### 3.1. Three-Dimensional (3D) Bioprinting Techniques and Their Working Principle

**Figure 3:**
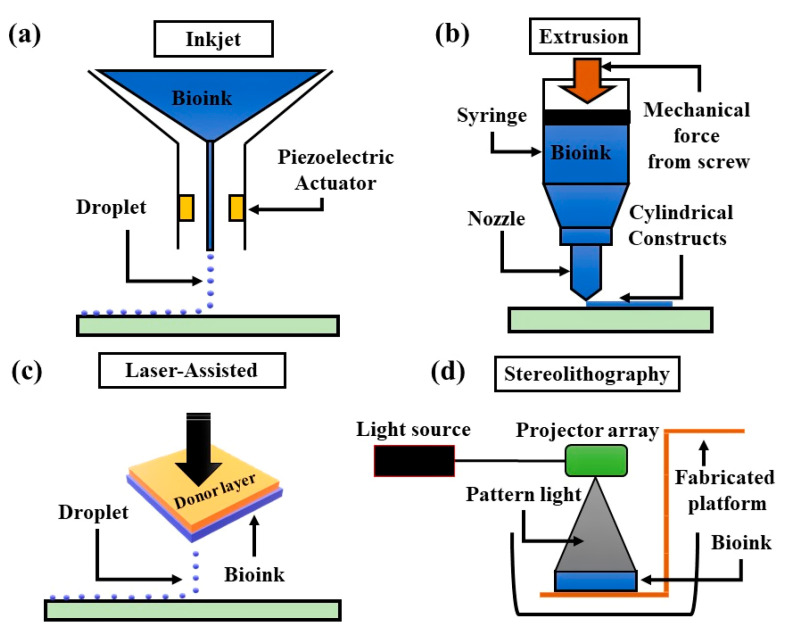
Four principal categories of bioprinting techniques. (**a**) Inkjet bioprinting involves deposition of bioink droplets through piezoelectric actuator. (**b)** Extrusion bioprinting uses mechanical force to generate and deposit continuous cylindrical stream of bioink. (**c**) Laser-assisted bioprinting uses energy-absorbing donor layer that responds to laser stimulation, a bioink layer underneath the donor layer, and a collecting layer to form tissue constructs. (**d**) Stereolithography bioprinting uses photosensitive bioink cured using precisely controlled light exposure projecting patterned binary image.

Three-dimensional (3D) bioprinting techniques can be divided into four categories based on their working principles: inkjet-based, extrusion-based, laser-assisted, and stereolithographic (Figure 3). Three-dimensional printing is based on the principle of layer-by-layer distribution of bioactive compounds, biochemicals, and specific cell lines, including the precise functional material distribution onto defined 3D structures (Table 5, Table 6 and Table 7).

#### 3.1.1. Inkjet-Based 3D Bioprinting

An inkjet-based 3D bioprinter primarily works by discharging liquid droplets onto a substrate under the influence of thermal and acoustic pressure. Thermal inkjet 3D bioprinting is realized by electrically heating the printer nozzle to produce pressure, which in turn produces droplets that are released from the nozzle. In contrast, acoustic-inkjet 3D bioprinting utilizes piezoelectric crystals to develop acoustic waves within the printer head that converts the liquid into droplets. In this process, if a voltage is supplied to the piezoelectric material, it undergoes a rapid structure change. As a result, sufficient pressure is required to push the droplets out from the nozzle. However, as these technologies have advantages and disadvantages, emerging technologies with a preference for inkjet bioprinting must be appropriates for desired targets.

Inkjet bioprinting is the deposition of low viscosity bioink onto a substrate in extremely small volumes (i.e., 1–100 picolitres) [78]. The principle of bioprinting is that a liquid biomaterial is printed layer-by-layer until the entire object is constructed. The liquid biomaterial is solidified rapidly to pass the print head while maintaining its construction structure. In this method, a crucial factor to adapting a biomaterial for bioprinting is to transform it from sol to gel. Polymers or composites are widely used as they can be polymerized using several methods, allowing them to be “3D-printed”. This bioprinter can be utilized in two different modes of operation: drop-on-demand and continuous inkjet bioprinting. In a thermally induced inkjet 3D printer, the bioink droplets are produced by heating and evaporation of the printing site, where bioinks form a vapor bubble that will multiply bioink droplets immediately upon discharge through the printing nozzle head drop by drop, forming a layer onto a substrate to fabricate the structures [79].

#### 3.1.2. Extrusion-Based 3D Bioprinting

Extrusion-based 3D bioprinting, also known as micro-extrusion bioprinting, is the most popular printing technique used in fabricating non-biological 3D structures. However, as the focus of several popular academic studies, this bioprinting technique has been utilized to fabricate 3D tissue or organ structures to enhance the regenerative potential of tissues and organs. These printers precisely control the temperature during material processing and distribution, as well as a platform that can move along the x-, y-, and z-axes. A fiberoptic light source is incorporated into this technology to highlight the discharge of the hydrogel bioink site where hydrogels are crosslinked with an activated photoinitiator. A few microextrusion bioprinters incorporate several printing heads that permit simultaneous sequential distribution of different materials.

Extrusion-based 3D bioprinting is applied in two different methods to achieve the targeted outcomes of tissues or organs: semi-solid extrusion (SSE)-based 3D bioprinting and fused deposition modeling (FDM)-based 3D bioprinting. In the SSE-based 3D bioprinting approach, the application of a highly compressed airflow is controlled by a rolling screw gear. It functions by the stable outflow of air streams throughout the nozzle with hydrogel bioinks discharged in a layer-by-layer manner, and the 3D structure of the construct is fabricated subsequently. In contrast, the FDM-based 3D bioprinting approach utilizes an extremely high temperature to melt the thermoplastic filaments. Subsequently, the bioinks are discharged layer-by-layer from the nozzle to fabricate a 3D structure. Extrusion and positioning technologies have evolved into the two principal components of extrusion-based 3D printers. These technologies must be highly precise to create visually and geometrically perfect structures.

#### 3.1.3. Laser-Assisted 3D Bioprinting

Laser-assisted 3D bioprinting functions by discharging bioinks with specific cells and biomolecules onto the surface using a laser as an energy source. A laser-assisted 3D bioprinter primarily includes a laser pulsed beam with laser targeting technology, a supported ribbon for donor transportation, and a biomaterial layer formed inside a liquid solution with recipient substrates towards the projector; hydrogels, cells, culture media, proteins, and even ceramic materials with biomaterials can be utilized during the laser-assisted bioprinting process. These bioprinters have a medium speed, and the process maintains approximately 90% of the viable cells. Laser-assisted bioprinting can fabricate tissue structures using pulmonary artery endothelial cells, human dermal fibroblasts, and even breast cancer cells; it was used to fabricate cellularized skin structures with appropriate cell densities in a multilayer tissue structure.

Throughout 3D bioprinting, the laser pulse concentrates on a specific region of the uppermost donor layer, which evaporates due to energy absorption, forming an air bubble with high pressure that is in contact with both the donor and bioink layers. The suspended bioink is forced by an air bubble to produce a droplet that is finally collected through the bottom collecting layer; as a result, a 3D tissue structure is constructed in a droplet-by-droplet manner. Relatively high concentrations and viscous materials are both suitable for laser-assisted bioprinting. Moreover, due to the short duration of a laser pulse, cells retain significantly higher cell viability of over 95%.

#### 3.1.4. Stereolithographic 3D Bioprinting

Stereolithography 3D bioprinting is a nozzle-free technology for creating 3D structures from biological and non-biological materials. Stereolithography technology facilitates precise construction and can utilize a wide range of materials. This technology involves light-sensitive hydrogels deposited layer-by-layer to fabricate a 3D structure. This technique has high speed (approximately 40,000 mm/s) and cell viability greater than 90% and has been utilized in various ways to develop organs and tissues in many species, especially humans; moreover, this technique was evaluated using DNA. The stereolithography technique is dependent on the solidification of a liquid photosensitive polymer upon irradiation and applies digital micromirror array technology to control the intensity of light that is sensitive to polymer materials. The photochemical solidification of polymeric materials leads to the fabrication of layers, which are combined to form the 3D structure.

**Table 5:** Three-dimensional (3D) bioprinting technologies in tissue engineering.

Bioprinting Method	Inkjet 3D Bioprinting	Extrusion 3D Bioprinting	Laser-Assisted 3d Bioprinting	Stereolithographic 3D Bioprinting
Description	Thermal, piezoelectric, or electromagnetic forces expel successive drops of bioink onto a substrate	Mechanical or pneumatic forces dispense bioink through a nozzle	Bioink and cells are suspended on the bottom of a ribbon and when vaporized by the laser pulse, are propelled to a receiving substrate	Use digital light to cure bioink in a layer by layer fashion
Advantages	High speed, availability, low cost	Ability to use high viscosity bioink and print high cell density	High degree of precision and the resolution, ability to use high viscosity bioink and print high cell density	High degree of fabrication accuracy, and low printing time
Disadvantages	Lack of precision in droplet placement and size, need for low viscosity bioink	Distortion of cell structure	Time consuming, high cost	Use of high-intensity UV light, lengthy postprocessing, lack of compatible materials
Effect on cells	>85% cell viability [79]	40–80% viability [79]	>95% cell viability [79]	>90% cell viability [79]
Cost	Low	Medium	High	Medium

**Table 6:** Comparison of 3D bioprinting modalities.

Factors	Inkjet-Based 3D Bioprinting	Extrusion-Based 3D Bioprinting	Laser-Assisted 3D Bioprinting	Stereolithographic 3D Bioprinting
Ink viscosity	3.5–12 mPa/s	Up to 6 × 10 mPa/s	1–300 mPa/s	No limitation
Cell density	Low, <10^6^ cell/mL	No limitation	Medium, <10^8^	No limitation
Resolution	High	Moderate	High	High
Print speed	Fast	Slow	Medium	Fast
Cost	Low	Medium	High	Low

**Table 7:** Human 3D tissues obtained from pluripotent stem cells by undifferentiated iPSCs-based 3D-bioprinting. (BJ fibroblast: Derived from BJ cells of human fibroblast; hiPSCs: Human induced-pluripotent stem cells; iChons: Induced chondrocytes; NFC: Nanofibrillated cellulose; HA: Hyaluronic acid).

Printing Methods	Printer	Diameter of Nozzle	Bioinks	Crosslinker	Cell Source	Lineage	Function	Ref.
Undifferentiated iPSCs-based 3D-bioprinting								
Extrusion based	Felix 3.0	40 µm	Geltrex	None	Custom-made fibroblasts derived hiPSCs	Plurilineage	3-germ layers	[80]
Extrusion based	3D Bioploter Envision TEC	200 µm	5% *w*/*v* alginate, 5% *w*/*v* carboxymethyl-chitosan, 1.5% *w*/*v* agarose	CaCl_2_	hiPSCs	Plurilineage	3-germ layers (neural tissue)	[81]
Extrusion based	3D Discovery regenHu	300 µm	Nanofibrillated cellulose (NFC) alginate (60:40) NFC with HA	CaCl_2_ (for alginate) H_2_O_2_ (for HA)	Custom-made hiPSCs, iChons	Cartilage	Pluripotency, Chondrocytes	[82]
Extrusion based	Custom-built	260 µm	2% *w*/*v* hydroxypropyl chitin (HPCH), 0–30% Mattrigel	Temperature 37 °C	hiPSCs from human peripheral blood mononuclear cells (hPBMC)	Plurilineage	Pluripotency	[83]
Laser assisted	Nd:YAG 1064 laser	N/A Droplet volume 0.01–1 nL	1 wt% HA Matrigel	-	hiPSCs	Cardiac	3-germ layers	[84]

## 4. Biofabrication Factors Pertaining to the Use of iPSCs Applied with 3D Bioprinting

### 4.1. Structural and Biological Biomimicry

In the 3D bioprinting process, the design and fabrication of specific architectures of tissues and organs requires an in depth understanding of native tissues and organs. Bioprinting engineered tissues/organs that are biomimetic and functional is a conceptually complex process. It is extremely difficult to recreate all the factors, including physical, chemical, and biological constituents, that shape the specific targeted tissues. The sheer volume and nature of cellular dynamic interaction required for even a simple tissue makes the fabrication exceedingly complex. This complexity is contingent upon the type of cells, immunological and biochemical factors such as signaling molecules, and environmental factors such as temperature, pressure, and electrical forces that must be considered [85,86,87]. Fabricated tissues have a significantly more complex structure and function owing to their 3D geometrical shape and the integration of mechanical forces. To minimize these complexities, different methods are utilized in the biomimetic approach to design and bioprint objects. In particular, a scaffold material that is essential in the bioprinting process is utilized to minimize the complexities in fabrication. Using a specifically optimized scaffold fulfills the structural and mechanical requirements of the targeted tissues. The selection of a specific scaffold is largely influenced by signaling pathways through the cells that interact with the extracellular matrix (ECM) component [88].

The application of bioreactors to regulate environmental factors is essential to the appropriate design and 3D bioprinting of tissues that have shown biomimetic functions. The bioreactors create a feasible environment or cellular microenvironments that mimic specific cells and tissues [89]. Bioreactors are regulated by different combinations of chemical, mechanical, and electrical requirements that encompass a specific 3D-cell culture [88]. These diverse combinations and their requirements also change over time to develop a specific favorable environment that allows for sequential cell maturation [90]. Various scientific studies have reported that several types of cells and tissues are not supported by their environments as certain external forces are required to provide important signaling cues that promote the appropriate development of cells or tissues. A bioreactor is an ideal mechanism that effectively facilitates these fundamental dynamic interactions between cells, tissues, and microenvironments. Most published reports demonstrate that bioprinting does not end with tissue and organ printing, and typically requires maturation time for specific cells. At this stage, bioreactors may be employed to support the bioprinting process towards effective biomimetic tissue development.

### 4.2. Bioink Preparation with iPSCs

**Figure 4:**
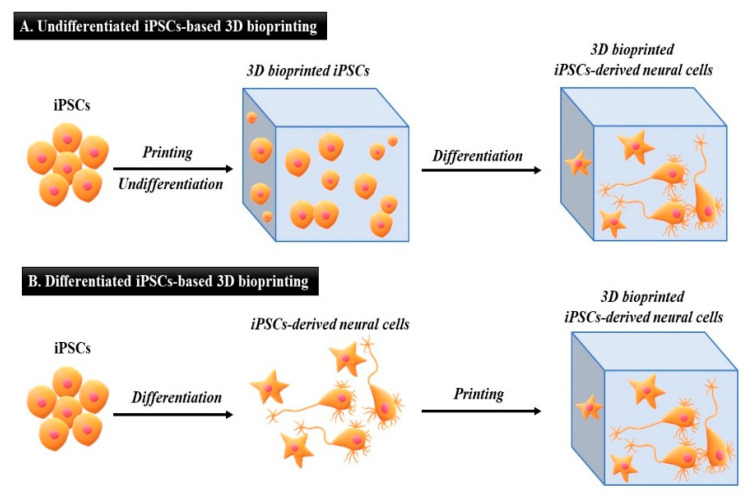
Three-dimensional (3D) bioprinting and differentiation of human-induced pluripotent stem cells (hiPSCs). Schematic representation of undifferentiated iPSCs-based 3D bioprinting and differentiated iPSCs-based 3D bioprinting strategies. As an example, neural tissue derived from human iPSCs is represented. (**A**) In undifferentiated iPSCs-based 3D bioprinting, hiPSCs are first printed to generate a 3D structure containing undifferentiated pluripotent cells. Subsequently, hiPSCs are induced to differentiate within the construct to obtain neural cells (e.g., neurons and astrocytes). In this case, there is no control on relative position and number of cells in final neural model. (**B**) In differentiated iPSCs-based 3D bioprinting, hiPSCs are first induced to differentiate to neural lineage with conventional culture methods. Then hiPSC-derived neural cells are bioprinted to generate the final neural model. In this case, cytoarchitecture of the neural model can be controlled.

Undifferentiated iPSCs could be encapsulated in bioinks to print 3D structures (Figure 4A). Specifically, iPSCs are induced to differentiate specific cell types within the final tissue structure. The microenvironment provided by the specific bioink is crucial for successful differentiation. Within such a 3D microenvironment, versatile and multipotent iPSC differentiation can be realized, which is not possible under 2D conditions. However, in this approach, the sensitivity of undifferentiated iPSCs to physical stress throughout the printing process would be a significant challenge [91,92]; therefore, the bioprinting process for undifferentiated iPSCs must be optimized.

**Figure 5:**
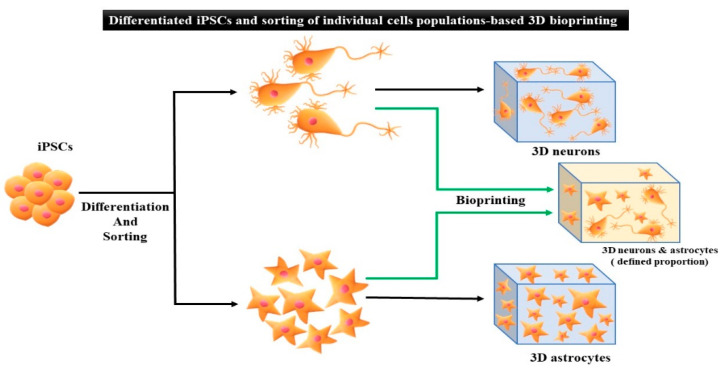
Differentiation of iPSCs and sorting of individual cell population-based 3D bioprinting. Schematic representation illustrating the possibility of controlling cell composition of 3D structure by differentiation of iPSCs, followed by sorting of individual cell populations and bioprinting. Cytoarchitecture and relative proportions of each cell type can be controlled. This approach also allows inclusion of other cell lineages, e.g., microglia, in the neural construct.

Conversely, in the differentiated iPSC approach (Figure 4B), iPSCs are differentiated into specific cell types [93]. Next, iPSC-derived specific cells are directly encapsulated in bioinks to print the final 3D tissue structures. In this study, the tissue or organ architecture composed of specific cell types (e.g., neuronal tissue) can be better manipulated when compared to the undifferentiated approach (Figure 5). Based on the intended purpose, the application of undifferentiated iPSC-based 3D bioprinting or differentiated iPSC-based 3D bioprinting should be prudently chosen. The following section reviews the various applications of undifferentiated iPSCs and differentiated iPSCs integrated with 3D bioprinting.

## 5. Applications of iPSCs-Based 3D Bioprinting

### 5.1. Undifferentiated iPSCs Generated by 3D Bioprinting

The major challenge in 3D bioprinting with undifferentiated iPSCs is the cellular sensitivity being affected by the mechanical force throughout the printing process. Therefore, bioprinting conditions should be precisely optimized. Furthermore, during the selection of iPSC-based biomaterials, various essential factors need to be considered in the formulation of bioink, including temperature, chemical and ionic concentrations, and light exposure (stress) at the time of crosslinking.

**Figure 6:**
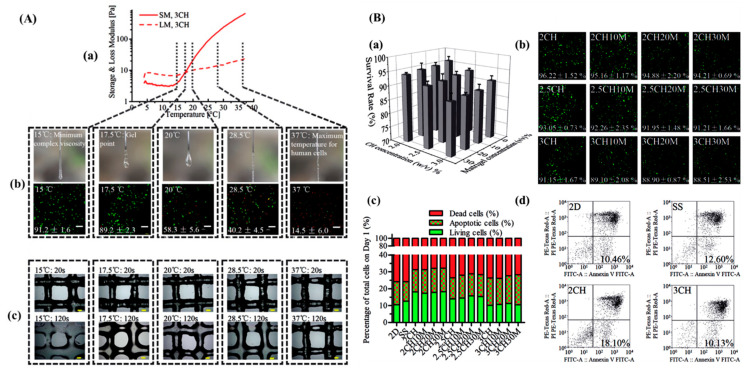
(**A**) Determination of temperature parameters when printing hiPSCs with hydroxypropyl chitin (HPCH)-based bioinks (for example, the following motion parameters for 3CH in this figure were set: nozzle diameter: 260 μm, extrusion and traveling speeds: 5 mm s^−1^). (**a**) The SM and LM values of 3CH hydrogel at different temperatures and five selected temperatures; 15 °C: the temperature at which hydrogel has minimum complex viscosity, 17.5 °C: gel point of this type of hydrogel, 37 °C: the highest temperature feasible for hiPSC culture. (**b**) The morphology when 3CH bioink was extruded from the nozzle at different nozzle temperatures and associated cell survival after extrusion. (**c**) The fiber morphology of printed 3CH grid structures immediately after printing and after crosslinking for 120 s at different temperatures. Scale bar: 200 μm. (**B**) Printing damage and survival rates of hiPSCs using different culture approaches. (**a**,**b**) Cell survival rates after 3D printing (day 0) using different bioinks. Scale bar: 200 μm. (**c**,**d**) Cell states (alive/apoptosis/dead) in different culture groups on day 1 (FITC (+) and PI (+): dead cells; FITC (+) and PI (−): apoptotic cells; FITC (−) and PI (−): living cells). Adapted from [83]. Copyright 2018 Biofabrication.

Currently, drop-on-demand, extrusion, and laser-assisted methodologies are used to print undifferentiated iPSCs (Table 7). An earlier study proposed the development of bioinks at different temperatures with undifferentiated iPSCs. The bioink was formulated with a chitin-based HPCH material combined with Matrigel for 3D printing of undifferentiated iPSCs. This bioink exhibited excellent printability at different temperatures (15–37 °C). Additionally, within a period of 24 h, more than 75.84% of cells died in the 2D culture group; in the 3D cell printing group, a significantly higher average of 17.87% of living cells were obtained using 2% (*w*/*v*) HPCH (2CH, 2CH10M, 2CH20M, and 2CH30M) (Figure 6) [83]. 

**Figure 7:**
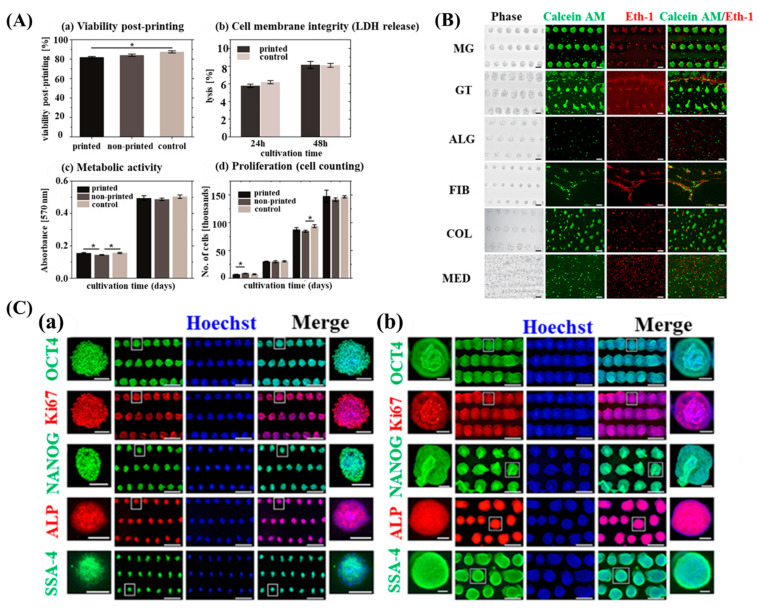
(**A**) Comparative evaluation of printed and non-printed hiPSCs by utilizing medium based approach without sols and gels. (**a**) hiPSCs viability was evaluated after 2–3 h of printing: printed hiPSCs viability (82 ± 1%), non-printed hiPSCs viability (82 ± 1%) and control cells (87 ± 1%). (**b**) Cell death of hiPSCs was observed after 48 h by evaluation of the released lactate dehydrogenase (LDH) into supernatant medium (24–48 h). (**c**) Evaluation of hiPSCs mitochondrial (metabolic) activity after printing (1–2 days) was significantly optimized as observed by MTT. (**d**) Proliferation was observed to be significantly similar in printed and non-printed hiPSCs (4 days) when compared with control cells. (**B**) Evaluation of hiPSCs within 3D bioprinted patterns on different gels including Matrigel (MG), Geltrex (GT), alginate (ALG), fibrin (FIB), collagen (COL) on hyaluronic acid and culture medium OR similarly complete E8 medium (MED) coated on glass plates. (**C**) Evaluation of tissue-like structure formation after printing to maintain pluripotency within medium and hyaluronic acid on Matrigel. (**a**,**b**) Immunostaining with pluripotency markers OCT4, NANOG, SSEA-4, and ALP and with proliferation marker Ki67 (1 and 6 days) to re-examine hiPSCs retention of proliferation and pluripotency. Adapted from [94]. Copyright 2018 Biofabrication.

Another study used laser-based 3D bioprinting. During the bioink formulation, undifferentiated iPSCs were combined with different biomaterials, including Matrigel, Geltrex, alginate, fibrin, and collagen, and suspended in a complete E8 medium to develop a unique bioink. The viability of hiPSCs in the printed group was over 82% compared to that of the non-printed group, which had a decreased viability of over 84%, and the control group by over 87%. They also observed a higher number of dead cells (Eth-1 positive cells) under all printing conditions. Throughout the printing processes, the hiPSCs showed a significant differentiation potential and pluripotency when HA, combined with E8 medium on Matrigel, was arranged in specified patterns (Figure 7) [94].

**Figure 8:**
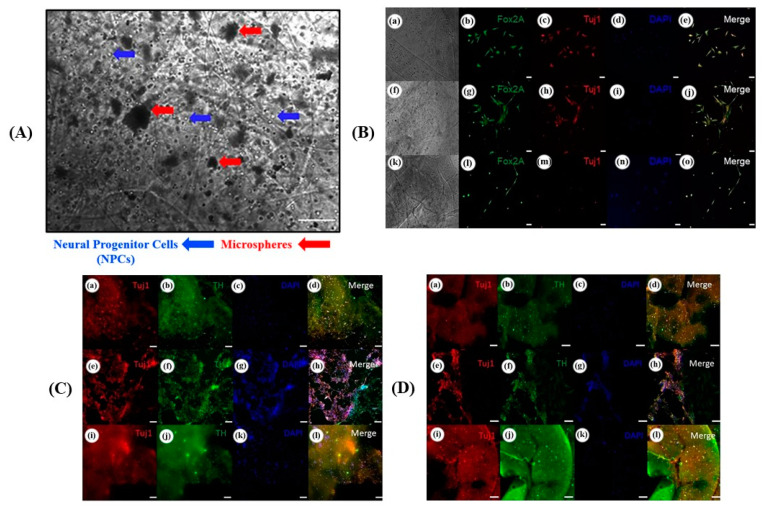
Application of 3D bioprinting of neural tissue structure. (**A**) Phase contrast images of day 0 printed structure showing neural progenitor cells (NPCs) and microspheres are dispersed throughout the fibers within the structures (100 µm). Top-down light microscopy image of bioprinted dome shaped structure consisting of NPCs with bioink containing encapsulated guggulsterone microspheres. (**B**) Immunocytochemistry was performed after 15 days of culture for the following markers: FoxA2 (a marker expressed by midbrain-type dopamine neurons shown in green), TUJ1 (an early marker for neurons shown in red), and the nuclear stain DAPI (4′,6-diamidino-2-phenylindole, shown in blue). (**a**–**e**) shows bioprinted tissues treated with soluble guggulsterone (SG), (**f**–**j**) shows bioprinted tissues containing unloaded microspheres (UM), and (**k**–**o**) shows bioprinted tissues containing guggulsterone microspheres (GM). (**C**) Immunocytochemistry was performed after 30 days of culture on cells that migrated out of the bioprinted structures for the following markers: TUJ1, TH (a dopaminergic neuron marker shown in green), and the nuclear stain DAPI. (**a**–**d**) shows bioprinted tissues treated with SG, (**e**–**h**) shows bioprinted tissues containing UM, and (**i**–**l**) shows bioprinted tissues containing GM. (**D**) Immunocytochemistry was performed after 30 days of culture on the cells embedded in different layers of bioprinted structures for the following markers: TUJ1, TH, and the nuclear stain DAPI. (**a**–**d**) shows bioprinted tissues treated with SG, (**e**–**h**) shows bioprinted tissues containing UM, and (**i**–**l**) shows bioprinted tissues containing GM. The scale bar is 100 μm. Adapted from [95]. Copyright 2019 Front. Bioeng. Biotechnol.

The fundamental advantages of 3D bioprinting technology are that it can be utilized for cost-effective production, customized to various geometrical shapes and sizes, and has negligible unit-to-unit variability when compared to other technologies. Therefore, 3D printing is expedient in designing and fabricating different types of drug delivery application for therapeutic purposes, as well as the engineering of artificial tissues and organs for biomedical applications [84]. Recently, the development of a methodology for drug delivery using 3D bioprinting technology with undifferentiated iPSCs has been successful. This is based on a supportive bioink combined with drug-delivering microspheres for bioprinting undifferentiated hiPSCs derived from neural progenitor cells. Cell viability on the first day of printing was more than 90%, while at 7 days post-printing cell viability was significantly increased to over 95%, and the gene expression of neuronal markers was enhanced as well (Figure 8) [95].

### 5.2. Differentiated iPSCs Generated by 3D Bioprinting

**Figure 9:**
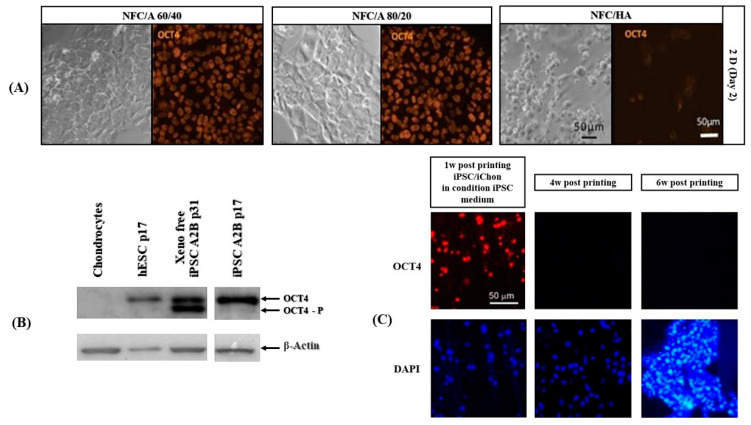
Application of 3D bioprinting in iPSCs-based cartilage tissue engineering with a nanocellulose/alginate Bioink. (**A**) Bright-field and fluorescent images at day 2 of iPSCs being in contact with three bioink compositions: (1) nanofibrillated cellulose (NFC)/A 60/40 crosslinked with 100 mM CaCl_2_ solution, (2) NFC/A 80/20 crosslinked with 100 mM CaCl_2_ solution, and (3) NFC/HA crosslinked with 0.001% H_2_O_2_ solution (the scale bars represent 50 μm). Cell morphology and Oct4-positive staining (orange) indicated compatibility and inertness of both NFC/A treatments. However, NFC/HA treatment changed cell morphology to be spherical with less Oct4 staining and fewer cells. (**B**) Western blot of cells before printing in primary chondrocytes passage 1 before irradiation in hESCs (human embryonic stem cell line SA121 passage 17) and in chondrocyte-derived iPSC line A2B in DEF xeno- and feeder-free passage 31 or DEF feeder-free culture at passage 17. No expression of pluripotency markers was detected in chondrocyte cultures. β-Actin was used in western blot analysis to show equal loading. (**C**) Immunohistochemistry for Oct4 and nuclei DAPI 1, 4 and 6 weeks after printing the iPSCs with iChons and maintaining samples in iPSC medium for first week, followed by induction of chondrogenic differentiation (the scale bar represents 50 μm). Adapted from [82]. Copyright 2017 Springer Nature.

Three-dimensional (3D) printing of differentiated iPSCs enables specific applications as the cells can be manipulated before the printing process (Table 8) [96]. Cartilage tissue structures from human-derived iPSCs with irradiated human chondrocytes (iCHons) that mimic native cartilage were fabricated by 3D bioprinting methods using nanofibrillated cellulose (NFC) composite-based bioink. This bioink was formulated using two types of bioink compositions: utilizing NFC with alginate (NFC/A) or hyaluronic acid (NFC/HA) and NFC/A (60/40, dry weight % ratio); noteworthy results were observed when NFC/A (60/40) bioink was utilized. Pluripotency was maintained for five weeks, confirmed by collagen type II expression and OCT4 tumorigenic gene expression (Figure 9) in the hyaline-like cartilaginous tissue [82]. 

Xu et al. [97] created iPSCs with a significant modification to the genes to improve the expression of bone morphogenetic protein 2 (BMP2). To this end, calcium phosphate-based scaffolds were built to enhance osteoconductivity and osteoinductivity, supporting the overexpression of BMP2 in iPSC-MSCs that increased the differentiation of osteocytes as the cells were cultured onto RGD functionalized with calcium phosphate scaffolds.

**Figure 10:**
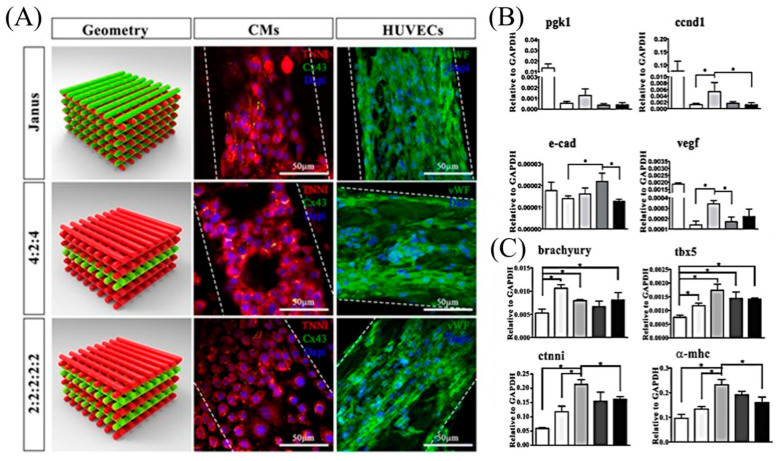
Applications of multicellular approach to 3D bioprinting by utilizing HUVECs and iPSC-derived cardiomyocytes to fabricate vascularized heart tissue engineered in alginate (ALG) and polyethylene glycol monoacrylate-fibrinogen (PF)-based bioinks. (**A**) Representative images showing TNNI (red) and Cx43 (green) expressions in CMs and vWF (green) labelling in HUVEC, after 7 days of culture, printed in three different spatial geometries. Janus structures contained the two different cell lineages within each laid fiber; 4:2:4 and 2:2:2:2:2 structures were printed altering two layers of HUVEC with two or four layers of CM. Scale bars represent 50 μm. (**B**) Relative gene expression relating to angiogenesis (pgk1, ccnd1, e-cad, vegf). Error bars represent ± SEM. Student’s *t*-test, * *p* < 0.05; N = 4. (**C**) Gene expression in vivo. Relative gene expression related to cardiac early genes (brachyury, tbx5) and cardiac late gene (ctnni, α-mhc) in 3D printed multi-cellular structures compared to CM in bulk structures and 3D bioprinted CMs in vivo. Error bars represent ± SEM. Student’s *t*-test, * *p* < 0.05.; *N* = 3. Adapted from [99]. Copyright 2017 Springer Nature.

Vascularized heart tissue was developed using iPSC-based 3D bioprinting methods [98]. Specifically, the development of functional heart tissue was implemented by 3D printing of iPSC-CM (8 × 10^6^ cells/mL) with HUVECs (6 × 10^6^ cells/mL) embedded in formulated hydrogel bioink containing 4% alginate and 1% PEG-fibrinogen; the cardiogenic markers were highly expressed in the fabricated structures. Immunofluorescence analysis was performed in multicellular tissue structures after 7 days of culture; HUVECs formed a monolayer, and mature CMs formed a large structure of endothelial-like cells with a diameter of 100 μm organized spatially (Figure 10A) [99].

In vitro qRT-PCR analysis showed that genes related to angiogenesis were significantly expressed on day 7. Moreover, vascular endothelial growth factor receptor 2, vascular endothelial growth factor, cyclin D1 (ccnd1, measuring the proliferation index), and B-cell lymphoma 2 gene expression (related to apoptosis) were significantly higher in the 4:2:4 and 2:2:2:2:2 geometries. On day 7, enhanced reendothelialization and proliferation were observed in all three geometries, indicating that apoptosis was prevented by vascularization. In the 4:2:4 structures, the expression of e-cadherin (e-cad) was much greater than that in the 2:2:2:2 structures in a larger sample (Figure 10B). In vivo qRT-PCR analysis revealed that this geometry enhanced the CM maturation by expressing late-specific cardiac genes, including cardiac troponin-I (ctnni) and alpha myosin heavy chain (α-mhc) (Figure 10C) [99].

A recent study demonstrated that 3D printing of cardiac patches consisting of omental cells, reprogrammed into iPSCs and subsequently differentiated into CM or EC, can be encapsulated with ECM-based bioink. In this study, omental tissue was obtained from the patient’s biopsy to extract the omental stromal cells and then utilized to develop decellularized ECM bioink. Moreover, functional vascularized patches can be fabricated using a 3D printing technique similar to the anatomy of the patient by using a supporting medium consisting of xanthan gum, sodium alginate, and calcium carbonate [101].

## 6. Disease Modeling on iPSCs-Based 3D Bioprinting Technology

The most advantageous aspect of using induced pluripotent cells in clinics is the ability of reprogramming of autologous cells taken directly from patients. At present, the majority of disease-modeling studies make use of traditional 2D cultures. Any monogenic or polygenic disease conditions can be re-created in such a 2D cell culture system. However, they possess several limitations including lack of heterogenic cell environment and the cell-to-cell communication cues. 3D disease models would help in understanding the disease mechanism in detail in the early stages of the disease. In this section, two representative examples of iPSCs-based disease modeling in cardiac and neurogenerative diseases is briefly discussed.

### 6.1. Cardiac Disease

Cardiovascular diseases remain the leading cause of death and account for more than 30% of all deaths. Harvesting cardiac tissue from patients with disease mutations for genetic studies is a highly challenging procedure. For this reason, iPSCs derived from the peripheral tissues of patients with disease specific mutations are a promising tool with which to study the cardiac pathophysiology and drug development. Cardiac tissues were biofabricated using hydrogels and supporting cells such as cardiomyocytes, endothelial cells, smooth muscle cells, and fibroblasts [108,109]. More recently, iPSC-derived organ-on-chips are used for modeling various diseases, including dilated cardiomyopathy as well as kidney glomerular injury [110,111].

### 6.2. Alzheimer’s Disease (AD)

AD is a progressive neurodegenerative disorder characterized by loss of cognition and disruption of basic functions, such as swallowing, walking, attention, and memory [112,113]. In recent years, it has become clear that multiple different brain cell types contribute to AD progression. Therefore, examination of their interactions and impacts on each other are of critical importance. Since the iPSCs can be differentiated into neural crest or neural progenitor cells [114], 3D bioprinted AD models will facilitate the development of effective therapeutics to combat AD-induced dementia.

## 7. Challenges and Future Direction Associated with iPSCs-Based 3D Bioprinting Technology

In spite of the challenges of the use of iPSCs and issues of 3D bioprinting, the potential of iPSC-based 3D bioprinted tissues/organs is tremendous in the regenerative medicine field. Yet, several important perspectives of bioprinting iPSCs should be considered as follows. Firstly, tissue-specific bioinks should be more prudently formulated for bioprinting-based iPSCs applications. future bioinks with tunable biomechanical properties that mimic the native tissue ECM would provide a deeper understanding of cell-bioink interactions and therefore the molecular pathways would have a major effect on the differentiation of the bioprinted iPSCs. Secondly, novel bioprinting strategies should be developed to minimize harmful effects on cells. Because iPSCs are sensitive, unlike cancer cell lines, several factors (e.g., mechanical, thermal, chemical stresses) caused during 3D bioprinting processes might result in cell-phenotype changes and functionality. Finally, optimal bioreactor systems should be integrated for accelerating the maturity and maintaining long-term microenvironment. That is, development of suitable post-processing strategies become necessary.

Organ-level systems in our body is highly sophisticated and thick. It requires adequate vascularization and enervation to allow for the development of biocompatible functions [115]. However, we have very limited capacity to recapitulating organ systems by biomanufacturing process. In parallel to the support to such structural recapitulation, stem cells, such as human iPSCs, have driven a paradigm shift in tissue regeneration and the modeling of human disease, and represent an unlimited cell source for organ regeneration and the study of human disease. Hence, more in-depth study on reprogramming patient-specific cells would be required to hold the promise of an enhanced understanding of disease mechanisms and organ-level regeneration [116]. 3D bioprinting has been successfully performed using multiple stem cell types of different lineages and potency. Additionally, more concentrated emergence of iPSCs with organ-on-chip technology enables the recapitulation of highly-predictable and reliable specific disease platforms [117], which must be also considered with 3D bioprinting technology.

## 8. Conclusions

The groundbreaking findings concerning iPSCs, primarily suggested by Prof. Takahashi and Prof. Yamanaka, have initiated a new direction of research into advanced 3D bioprinting and regenerative medicine. In this paper, we reviewed the generation of iPSCs and the advantages and disadvantages of using iPSCs in tissue engineering. Furthermore, several important considerations with respect to the integration of iPSCs with 3D bioprinting were examined. The applications were further divided into undifferentiated and differentiated iPSCs based on specific purposes. Finally, several potential applications were broadly introduced.

Compared with ECSs, iPSCs, which can be successfully generated from somatic cells by various transfecting factors (e.g., Oct4, Sox2, and K1f4), can overcome the limitations of mere multipotent stem cells that differentiate into only several lineage cells and the ethical concerns associated with ESCs. Therefore, utilizing iPSCs in stem cell therapy has a significant potential for applications in regenerative medicine. Moreover, extensive advances in 3D bioprinting have been increasingly in the spotlight. We envision that the combination of iPSCs and 3D bioprinting can make significant progress in future regeneration techniques that can eventually contribute to treating incurable diseases, such as Alzheimer’s and cancer.

## Figures and Tables

**Table 1:** Several factors and chemicals (small molecules) are able to replace the basal transcription factors (Oct4: Octamer-binding transcription factor 4; Sox2: SRY-Box transcription factor 2; Klf4: Kruppel like factor 4; c-Myc: c-myelocytomatosis oncogene product; N-Myc: (MYCN) v-myc avian myelocytomatosis viral oncogene neuroblastoma derived homolog, Nanog: Hoeobox protein NANOG-transcription factor, Lin28: Lin-28 Homolog A protein- transcription factor) used for reprogramming of cells.

Factors/Chemical (Small Molecules)	Function	Replacing Transcriptions Factors	References
Nanog	Embryonic stem cells (ESCs)-specific transcription factor	Together with Lin28, able of replacing Klf4 and c-Myc	[25]
Lin28	Embryonic stem cells (ESCs)-specific RNA-binding protein	Together with Nanog, able of replacing Klf4 and c-Myc	[25]
Esrrb	Orphan nuclear receptor	Klf4	[26]
SV40 LT (T)	SV40 large T antigen used for cell transformation	Klf4; N-Myc and Lin28, Nanog	[27]
BIX-01294	Inhibitor of G9a histone methyltransferase	Sox2, Oct4	[28]
VPA	Inhibitor of histone deacetylase	Klf4 and c-Myc	[29]

**Table 2:** Different cell sources and different combinations of reprogramming factors have been used by different groups for reprogramming to iPSCs (Oct4: Octamer-binding transcription factor 4, Sox2: SRY-Box transcription factor 2, Klf4: Kruppel like factor 4, c-Myc: c-myelocytomatosis oncogene product, Nanog: Hoeobox protein NANOG-transcription factor, Lin28: Lin-28 Homolog A protein- transcription factor).

Type of Cells	Reprogramming Factors	References
Fibroblast	Oct4, Sox2, Klf4, c-Myc	[30]
	Oct4, Sox2, Lin28, Nanog	[25]
Keratinocytes	Oct4, Sox2, Klf4, c-Myc	[31]
Cord blood endothelial cells	Oct4, Sox2, Lin28, Nanog	[32]
Cord blood stem cells	Oct4, Sox2, Klf4, c-Myc	[33]
Neural stem cells	Oct4	[34]
Melanocytes	Oct4, Sox2, Klf4, c-Myc	[35]
Amniotic cells		[36]
Adipose derived stem cells		[37]
Hepatocytes		[38]
Circulating T cells		[39]
Astrocytes		[40]
Peripheral blood		[41]
Kidney mesangial cells		[42]
Urine cells	Oct4, Sox2	[43,44]

**Table 3:** Different delivery methods for transfer of different combinations of transcription factors (Oct4: Octamer-binding transcription factor 4, Sox2: SRY-Box transcription factor 2, Klf4: Kruppel like factor 4, c-Myc: c-myelocytomatosis oncogene product) have different efficiencies of reprogramming.

Methods	Reprograming	Factors	Type of Cell	References
Integrating	Retroviral transduction Lentiviral	Oct4, Sox2, Klf4, c-Myc	Mouse fibroblast	[30]
		(Oct4, Sox2, Klf4, c-Myc) + (VPA)	Neonatal	[29]
		Oct4, Sox2, Klf4, c-Myc	Human fibroblast	[25]
	Inducible lentiviral	(Oct4, Klf4) + parnate + CHIR99021	Neonatal	[45]
		Oct4, Sox2, Klf4, c-Myc	Human fibroblast	[46]
Non-integrating	Sendai virus	Oct4, Sox2, Klf4, c-Myc	Human fibroblast	[47]
	Adeno viral transduction		Mouse fibroblast	[48]
	Plasmid DNA transfer		Fibroblast	[49]
	lox p lentivirus		Fibroblast	[50]
	PiggyBAC		Fibroblast	[51]
	Polyarginine tagged polypeptide		Neonatal fibroblast	[52]
	RNA modified synthetic mRNA		Human fibroblast	[53]

**Table 4:** Advantages and disadvantages associated with the application of iPSCs.

	Advantages	Disadvantages
Due to characteristics of iPSCs	Eliminates ethical issues	Premature aging
	Reduced chances of immunorejection [55]	High rate of apoptosis
	Differentiation to any cell type	Low rate of reprogramming
	Reduced risks of clinical trials	Low-level DNA damage repair [56]
	Consistent phenotypes for disease modeling [57]	Sensitive to ionizing radiation [58]
Due to technology of development	Possible preservation	Tumourogenesis [59]
	Continuous cell supply	Insertional mutagenesis [49,60]
	Possible preservation	Tumourogenesis [49]
	Availability and accessibility of source cells	Chances of development of diseases due to factors used [61,62,63,64]
	Personalization of treatment [65]	Suboptimal standardization [66]
Applications	High-throughput screening of drugs and toxicity prediction [67,68]	Complex assessment
	Reduced cost	Complex diseases become difficult to be modeled
	Gene correction therapies add to the benefits from iPSCs [65]	Immature cells cause problems during cell line development

**Table 8:** Bioprinting of iPSC-derived cells for cartilage, heart, hepatic, neural, and skin tissues. * AG/MC: alginate mixed with metacellulose; GMHA: glycidal-methacrylate-hyaluronic acid; NFC/A: alginate; NFC/HA: hyaluronic acid; OPC: oligodendrocyte progenitor cells; SNPC: iPSC-derived spinal neuronal progenitor cells.

Tissue	Cell	Bioinks	Cross-Linker	Printer	Ref.
Cartilage	hiPSC-derived chondrocytes	NFC/A * NFC/HA *	CaCl_2_	3D Discovery (regenHu, Switzerland)	[96]
	iPSC source: chondrocytes				
Heart	hiPSC-derived CM, SMC, EC	GelMA	† Multiphoton excitation	Custom-built multiphoton laser-scanning 3D printer	[100,101]
	iPSC source: cardiac fibroblasts				
	HUVEC and iPSC-CM	Alginate and PEG-fibrinogen hydrogel	CaCl_2_ and UV	Custom designed MPH for the simultaneous extrusion of multiple bioinks	[99]
	iPSC source: mouse embryonic fibroblasts				
	CM and EC derived from the same iPSC	Decellularized omental tissue printed in supporting medium	37 °C for 45 min	3D Discovery (regenHu)	[102]
	iPSC source: omental stromal cells				
	iPSC-CM, HUVEC and NHDF	Scaffold free	-	Regenova (Cyfuse Biomedical K.K.)	[103]
	Human skin fibroblasts	Scaffold free	-	Novogen MMX (Organova)	[104]
Hepatic tissue	iPSC-HPC	GMHA *, GelMA	UV polymerization	Custom extraction based 3D printer	[105]
	iPSC source: human perinatal and foreskin fibroblast				
Neural tissue	SNPC and OPC	Matrigel as cell laden bioink AG/MC * as supporting ink	Temperature, CaCl_2_ or BaCl_2_	Custom microextrusion-based 3D printer	[106]
	iPSC source: † UMN-X7 and UMN-3F10				
Skin	iPSC-derived endothelial cells	Alginate molds	CaCl_2_	† Object24 3D-Printer (Stratasys)	[107]
	iPSC source: human fibroblast from foreskin				

†: Indicates cells from the human-induced pluripotent stem cell (hiPSC) line.

## Data Availability

Data sharing not applicable.
